# Old-age Care Responsibilities in China’s National Five-year Plans (2001—2025): Towards Active Ageing and Explicit Familialism

**DOI:** 10.1007/s10823-026-09594-9

**Published:** 2026-08-24

**Authors:** Dongyang Yu, Virpi Timonen, Minna Zechner

**Affiliations:** 1 Faculty of Social Sciences, University of Helsinki, Unioninkatu 37, Helsinki, Finland; 2 School of Social Work and Social Policy, Trinity College Dublin, Dublin, Ireland

**Keywords:** Active ageing, China, Familialism, Old-age care responsibility

## Abstract

Rapid population ageing is challenging China’s old-age care (OAC) system. The traditional role of the family in caring for older adults has been declining due to decreasing family size and urbanisation. The Five-Year Plans for National Economic and Social Development (FYPs) of the People’s Republic of China provide a general framework for the direction of OAC policy. This article focuses on changes in OAC responsibilities among families, the government and individuals across five FYPs between 2001 and 2025. Content analysis indicates that policy in China is shifting from implicit familialism towards explicit familialism, with families remaining the primary providers of care while receiving indirect support in the form of tax relief. Alongside the indications of moving towards explicit familialism, the FYPs indicated a growing role for the government through expanding care provisions, integrating technology into OAC, improving older adults’ economic security, and promoting home- and community-based care. These policy developments expand care provisions beyond families as complements to family care, support financial independence in later life and indicate incipient de-familialism. Individuals are increasingly expected to work longer, engage in personal pension arrangements and consume OAC products and services, thereby assuming greater responsibility for their own OAC and reflecting policy directions consistent with active ageing.

## Introduction

Long-term care (LTC) refers to the provision of health, personal care and social services over an extended period of time to older people who experience difficulties in performing activities of daily living (Kane et al., 1987). In contrast to LTC, old-age care (OAC, yanglao or养老) is a broader concept that comprises both care and income security and conveys the moral value of filial piety. Traditionally, in China, OAC is seen as “a familial or inter-familial issue” (Liu & Sun, 2015) arising from the filial piety obligation that adult children should support ageing parents physically, financially, and emotionally (Zhan et al., 2011). Filial piety, the ideational foundation of OAC, emphasises children’s unconditional submission to parents, provision of daily care to ageing parents by adult children or arrangement of daily care and support for older family generation(s), and perpetuation of family lineage (Yan, 2016, 2023). A child who lives with and greets their parents both in the morning and in the evening traditionally is considered the role model of filial piety, although few children today adhere to this behaviour. As such, filial piety is a moral duty and OAC traditionally involved adult children living with or near their parents, caring for, respecting and supporting them, and ensuring their wellbeing (Cheung, 2019), regardless of the health situation of parents.

The economic reforms in the late 1970 s resulted in modernisation and urbanisation of China and modifications to the practices of filial piety and OAC (Cheung, 2023; Sun, 2017; Yan, 2016). The moral obligations of OAC have been reduced to older adults receiving support when they need it, regular visits and contact from children, and reunions during traditional festivals (Cheung, 2023). However, filial piety still plays a role in OAC. Nearly 14% of households still consisted of three or more generations living together by the end of 2020 (National Bureau of Statistics, 2021). OAC also goes beyond a moral obligation and has become a legal obligation in China. According to China’s law on rights and interests of older people, children must provide material support, mental comfort, companionship, and assistance with daily living for parents (The State Council, 2012).

China is currently experiencing significant and rapid demographic ageing. In terms of World Health Organisation’s (WHO) definition of ageing society, China became an ageing society in 2000 and an aged society in 2021. The share of people aged 65 and over in the population has increased from 7% to 14% between 2000 and 2021 (National Bureau of Statistics, 2001 & 2022). The number of older adults is expected to continue to rise until the end of this century (Zhu & Walker, 2022). The decreasing family size, due to the one-child policy that was in force between 1979 and 2015 (Cheung, 2019; Gong, 2020), urbanisation (Zhao et al., 2020) and large-scale internal migration (He & Ye, 2014; Liu & Cook, 2020), limit the capacity of families to care for ageing parents. Rural-urban migration has resulted in a growing proportion of older adults in rural populations (Chen et al., 2014; Yuan & Zhou, 2019). The left-behind older people are less likely to receive care from their children due to geographic distance (Zheng & Wu, 2022), making shortfall of OAC in rural areas more severe than in urban ones. However, families still take the main responsibility for supporting older adults (Chappell & Funk, 2012; Cui & Du, 2021; Lee, 2018; Song & Ji, 2020). The growing need for OAC and the declining traditional OAC model make it necessary to redistribute OAC responsibilities among families, the government, and individuals (Wang, Zhang & Peng, 2021). The issue of who should support older adults has gained increasing attention with the advent of an aged society in China at the beginning of the 21 st century (Lei & Yang, 2021).

Liu (2024) interviewed urban residents and revealed a reduction in the reported instrumental support from children to their parents across three generations. Despite the declining role of families in caring for older adults, China still mainly relies on informal care provided by family members (Liu & Cook, 2020; Su et al., 2022). Song and Ji (2020) pointed out that because of limited public care provisions, older adults have to rely on family care (see also Lee, 2018; Qi, 2021). The development of care homes and other formal OAC services complement the care provision by families. However, living in a retirement or nursing home is considered undesirable by many older adults as these facilities are associated with poor living conditions, inadequate care, and even bullying by staff (Ma et al., 2019). Entry into a nursing home can be perceived as a sign of children’s failure to fulfil filial responsibilities, which may be viewed as “losing face” (丢脸 or 没面子) (Ma et al., 2019) and hence most prefer to depend on their children (Su et al., 2022). The norms of familialism and filial piety still feature in OAC.

The 7th National Population Census report showed that 35% of Chinese older adults primarily rely on pensions for their living expenses, while 33% and 22% depend on economic support from family and income from their own work, respectively (National Bureau of Statistics, 2021). This report marks the first time that pensions have become the primary source of income for older adults (Du & Wu, 2023) and indicates the rising role of individuals generating their own incomes through employment and pensions, especially for older adults in urban areas of China. Approximately 70% of urban older adults depend on pensions to live, compared with 32% of rural older adults in China (National Bureau of Statistics, 2021). Only 17% of urban older adults depend on economic support from their families. In contrast, family economic support remains the main source of income for rural older people in China, and 37% of rural older adults are financially dependent on their families. Regardless of the different proportions of income sources between urban and rural older adults in China, compared to the 6th national census in 2010, both rely more on pensions and less on family members to financially support them (National Bureau of Statistics, 2001).

Against the background of the changes outlined above, there is a need to understand how the distribution of responsibility for OAC is constructed and steered through government policy. China’s National Five-Year Plans (FYPs), which are the most important policy blueprints, set medium- and long-term goals and policy guidelines for sustainable national economic and social development in the following five years. The strategies outlined in FYPs to address population ageing reflect how the Chinese government frames ageing and OAC responsibilities. The formulation of a FYP progresses through four stages: mid-term evaluation of the preceding plan’s mid-term implementation; framework development via national research incorporating field investigations based on mid-term evaluation; drafting grounded in stakeholder consultations; and formal outline compilation incorporating public input and legislative approval (Cyberspace Administration of China, 2016). The National Development and Reform Commission, coordinating ministries, research institutes, enterprises and experts in various fields, steers the FYP lifecycle. The planning undergoes multi-tiered reviews by the State Council and the Standing Committee of the Political Bureau, followed by preliminary examination by the National People’s Congress Financial Committee, before final deliberation and approval by the National People’s Congress. Lower levels of government are subordinate to planning at higher levels of government (Hu et al., 2022). FYP implementation deploys binding targets through ministerial and provincial task allocation, drives progress via resource distribution and merit-based incentives, and seeks to ensure accountability through mid-term evaluations and dynamic policy adjustments (Jiang et al., 2017; Liu et al., 2018).

The 1 st FYP was published in 1955 and the plans continue to be published every five years. The continuity of FYPs allows for the analysis of the historical evolution and changes in how the distribution of OAC duties is construed at the highest level of policymaking in China. The key characteristics of the FYPs, namely their policy orientation, continuity, and broad scope make them an ideal resource for studying the policy aims concerning the distribution of OAC responsibilities across time in China. The 10th Five-Year Plan, published in 2001, was the first one to mention population ageing as a policy-relevant concern, and hence the empirical material for this research starts in 2001 and ends with the most recent (2025) plan. This article aims to outline and explicate changes in the roles of families, the government, and individuals in caring for older adults in China in the Five-Year Plans spanning 2001 to 2025, and the emergence of active ageing in China’s policy discourse, drawing on the framework of varieties of familialism and on the concept of active ageing. Despite the increasing involvement of the non-governmental organisations (NGOs) and private (commercial) actors in OAC, they are not independent stakeholders due to their institutional constraints: NGOs in China are highly dependent on the government due to registration requirements, policy alignment mandates, as well as for funding and daily operation (Xu & Byrne, 2020; Chen & Hooimeijer, 2024). Profit-driven private sector operators predominantly target older adults with low level of care needs through premium services (Glinskaya et al., 2025), with costs largely borne by individuals and families (Maags, 2022).

## Varieties of Familialism

The concept of filial piety aligns with the norm of familialism in obeying, respecting and supporting one’s parents (Bedford & Hwang, 2003; Qi, 2016; Sabogal et al., 1987). Familialism refers to a value that prioritises family interests and prosperity over those of the individual, sometimes even requiring the individual to sacrifice personal rights and self-realisation for the benefit of the family (Garzón, 2000; Yan, 2018). Familialism also implies that family members take on caring functions and individuals are dependent on their family for care (Leitner, 2003). Conversely, de-familialism refers to declining reliance on family care and reduced dependency of individuals on their family (Leitner, 2014). It promotes the state, market, and non-governmental organisations, rather than the family, as the primary sources of care for older adults (Micho’n, 2008; Žalimienė et al., 2020). In terms of strong/weak (de-)familistic policies, Leitner (2003) outlines four ideal varieties of familialism, including explicit familialism, optional familialism, implicit familialism and de-familialism. A state in which policy facilitates family care for older adults and frames family care for older adults as an obligation is characterised as explicit familialism. Similar to explicit familialism, optional familialism supports family care but also offers options to choose between family care and alternatives to it. Implicit familialism neither strengthens nor weakens the family’s caring functions through policy and family ends up playing an essential role in OAC due to lack of alternatives. The de-familist model reduces older adults’ reliance on family care through provision of market and/or state-provided care. We utilise this framework to distinguish varieties of familialism in the FYPs of China between 2001 and 2025.

## Active Ageing

The concept of active ageing encourages individual independence and self-care in later life, highlighting the role of older adults in OAC. The framework of active ageing was first proposed in the Second World Assembly on Ageing in 2002 by the World Health Organisation (WHO). The active ageing perspective advocates that the aged population ought to have “continuing opportunities for health, participation, and security” (WHO, 2002). The ultimate goal is to enhance quality of life as people age (Ko & Yeung, 2019). The multidimensional ideation of active ageing challenges the perception that individuals in old age are passive and dependent (Dommaraju & Wong, 2021). It also acknowledges that individuals have potential and should have opportunities to actively engage in work and volunteering (Peng & Fei, 2013), thereby implicitly expanding their responsibility for their own wellbeing (Boudiny, 2013; Foster & Walker, 2015; Timonen, 2016). Compared to the wider WHO framework of active ageing, in EU policy discourse, active ageing focuses primarily on the extension of employment (Foster et al., 2021; Walker & Maltby, 2012). The Council of the European Union (2012) released guiding principles for active ageing in 2012, the first of which was to encourage older people to participate in the labour market and providing opportunities, such as access to skills training and lifelong learning, to ensure longer working lives. The concept of active ageing has been criticised because of its strong emphasis on productivism (Foster et al., 2021; Moulaert & Paris, 2013; Timonen, 2016). The role of active ageing as part of the policy response to population ageing in China is analysed by examining the shifts in the distribution of OAC responsibilities among families, the state, and individuals in FYPs between 2001 and 2025.

## Methods

To discern the general trends in policymaking concerning the distribution of responsibility in OAC, five FYPs in Chinese, namely the 10th Five-Year Plan (2001–2005), the 11th FYP (2006–2010), the 12th FYP (2011–2015), the 13th FYP (2016–2020), and the 14th FYP (2021–2025), were downloaded from the website of the State Council of the People’s Republic of China. The length of these five documents in Chinese ranges from 29,000 to 65,000 characters. The Chinese keywords 养老 (old-age care/elderly care), 老人 (older people), 老年人 (older people), and 老龄化 (ageing) were used to conduct a comprehensive search across the full texts of the Chinese versions of the five FYPs. All sentences containing these keywords were extracted for analysis. If keywords appeared in section headings, the entire section was included to preserve contextual coherence. The length of these selected passages ranges from approximately 208 to 500 characters in the original Chinese texts. Corresponding paragraphs from the English translations of the FYPs were then identified and extracted for comparison. The English versions of the 13th and 14th FYPs were retrieved from a legal information retrieval system launched by Peking University’s Legal Information Centre. Since there are no official English versions of 10th, 11th and 12th FYPs, the first author translated the corresponding Chinese texts into English, following the language style of the English versions of the 13th and 14th FYPs. The identified texts were located and selected from English version documents, along with the corresponding chapter and section names, as presented in Table 1 below. All the retrieved texts were reviewed again to assure the interpretations are correct and consistent.

**Table 1:** Selected Sections from 10th, 11th, 12th, 13th, 14th Five-Year Plans

Document	Chapter	Section
The 10th FYP (2001–2005)	18. Expand employment and improve social security system	2. Improve social security system 3. Develop other social security services
The 11th FYP (2006–2010)	6. Improve the appearance of rural areas	4. Develop rural social security system
	38. Carry out population work comprehensively	3. Actively respond to population ageing
	39. Improve people’s living standards	3. Enhance social security system
The 12th FYP (2011–2015)	16. Develop life service industry	3. Encourage the development of household services
	33. Improve social security system covering urban and rural residents	1. Improve social security system
	36. Carry out population work comprehensively	4. Actively respond to population ageing
The 13th FYP (2016–2020)	10. Open up new space for drivers of development	1. Upgrade in the structure of consumption
	24. Increase quality and efficiency within the service sector	3. Better consumer services
	35. Improve the housing supply system	2. The real estate market
	49. Improve the strategy for opening up	4. Foreign capital and outbound investment
	61. Provide more public services	2. Diversified public service needs
	64. Carry out social security system reform	1. Improve social insurance system 3. Support the development of social welfare and charity
	65. Actively respond to population ageing	1. Balanced population development 2. Improve old-age care services
	68. Provide more cultural products and services	2. Construct modern public cultural service system
The 14th FYP (2021–2025)	10. Development of the service sector	2. Development of consumer services
	12. Greater domestic circulation	1. Supply chain adaptability
	14. Strategies to boost domestic demand	1. Expanding consumption
	16. A digital society	3. A new blueprint for digital life
	45. A national strategy in active response to population ageing	3. Improve old-age care service system
	49. A multi-tiered social security system	1. Improve social insurance system

In total, 24 sections commenting on OAC relevant matters in the FYPs were identified, of which 2 sections in the 10th YFP, 3 sections in the 11th and 12th FYPs, 10 sections in the 13th FYP, and 6 sections in the 14th FYP. The number of sections related to OAC in FYPs therefore evinces an upward trajectory over time. The social security system and OAC related services are the most frequently outlined topics in the extracted sections. It is interesting to note that there are also aspects of the economy that subsume ageing-related issues and technology has emerged as the new tool for OAC.

### Limitations

While the policy orientation and continuity of five FYPs make them ideal for examining the development of policymaking over time, this article does have some limitations. The high-level policy documents only reflect broad policy directions, whereas the implementation at the local level often varies. For example, the gaps in resource allocation and OAC service coverage between urban and rural areas may result in policies being implemented more effectively in cities compared to in rural villages. Along with this, individuals from different socioeconomic backgrounds have unequal opportunities to benefit from these policies. Higher-income groups are more likely to financially prepare for their later years, whereas lower-income groups may struggle to build their economic security net. As such, the findings should be read as analysis of broad policy aims that are only partially and variably converted into actual policies.

### Data Analysis

The data were analysed by content analysis, which is suitable to identify patterns within data (Sgier, 2012) and enables distillation of a large number of texts into categories and concepts in a systematic way (Stemler, 2000). It does not only describe manifest texts but also allows interpretation of latent meanings in texts (Schreier, 2012). Lindgren and colleagues (2020) distilled content analysis process into three steps, namely selecting related segments, “condensing and coding”, and creating (sub)themes and (sub)categories. The first author conducted the coding process manually, identifying core concepts and grouping them into thematic categories. The second and third author cross-validated codes and refined thematic coherence. A three-tiered themed coding system was implemented to categorise responsibilities across stakeholders. Three main themes, the role of families in OAC, the role of the government in OAC, and the role of individuals in OAC were created: these constitute the three main sections in the findings. Eight sub-themes were identified and are outlined under the main section that they relate to in the findings. The sub-theme relating to the role of families is *supporting family care*; relating to the role of government, the sub-themes are *broadening the care system*, *developing OAC facilities and services*, *employing technologies for OAC*, and *improving pension schemes*; and relating to the role of individuals, the sub-themes are *encouraging private OAC consumption*, *extending working lives*, and *improving personal pension planning* (see Table 2).

**Table 2:** Main themes and sub-themes

Themes	Sub-themes	Examples from texts
Family responsibility	Supporting family care	*We will … promote supportive policies for family care of older adults*
Government responsibility	Broadening the care system	*We will establish a home-based*,* community-supported and institution-supported old-age care service system*
	Developing OAC facilities and services	*We will increase the number of community day care centres for the aged.*
	Employing technologies for OAC	*Design programs aimed at finding systematic technological solutions to addressing bottlenecks in …old-age care.*
	Improving pension schemes	*We will expand the coverage of urban basic pension insurance*,* gradually complete individual accounts.*
Individual responsibility	Encouraging private OAC consumption	*We will work steadily to promote spending on … old-age care.*
	Extending working lives	*We will … implement policies to gradually increase the retirement age.*
	Improving pension planning	*We will establish a multilevel pension insurance system that includes… commercial pension insurance.*

### The Role of the Government in OAC

#### Broadening the Care System

Three FYPs outlined the Chinese government’s efforts to develop the care system. During the 12th FYP (2011–2015), the framework of “home-based care as core support, community-based care as necessary support, and residential care as supplementary support” was introduced (Chap. 36, Sect. 4). In the following FYP (2016–2020), this approach evolved into advocating the merging of “home-based care” and “community-based care” into “home and community-based care” (Chap. 65, Sect. 2). The emphasis on home and community-based care remained consistent. The positioning of care institutions as supplementary support in care system reflects Chinese older adults’ reservations about institutional care (Guo, 2019). The 14th FYP (2021–2025) highlighted home-based care, older adults receiving home delivery services, and community-based care, where they stay at community care centres during the day and return home at night. Institutional care providers were encouraged to provide care services for older adults in the community (Chap. 45, Sect. 3). Home care services (for example, personal care services such as eating, bathing, toileting, washing, and dressing) support older adults living in their own homes and allow them to reduce their reliance on family care and to age actively within their own homes. By transferring care duties from families to state-monitored community services (Chen & Hooimeijer, 2024) and subsidised institutions, the promoted OAC system supports older adults’ independence and reconfigures traditional familial care expectations, reflecting the idea of active ageing and emergent de-familialisation.

#### Developing OAC Facilities and Services

The development of public and private OAC facilities and services was stressed in four FYPs. From 2001, the Chinese government began to “strengthen the construction of services and facilities for older adults and develop public projects and the market of ageing” (10th FYP (2001–2005), Chap. 18, Sect. 3). The idea of actively enhancing public OAC facilities and initiating a government-led OAC market was emphasised in the 12th FYP (2011–2015), and the Chinese government set the target of “achieving a ratio of 30 old-age care beds per 1000 older adults” (Chap. 36, Sect. 3). The 13th FYP (2016–2020) continued to expand OAC options by “building public services and facilities for older adults”, “opening older adults care market, and … supporting all types of private capital in providing more old-age care products and services” (Chap. 65, Sect. 2). The market-driven OAC was first introduced in the 13th FYP. In the following FYP (2021–2025), “the government-enterprise joint programme” for OAC was put forward, “where the private business sector was encouraged to run government-funded care institutions for older adults” (Chap. 45, Sect. 3). The Chinese government actively created an OAC market for private businesses at an early stage and then shifted to increased private sector involvement in OAC. The development of OAC market increases the supply of OAC products and services that serve as alternatives for family care, indicating an emerging tendency towards de-familialism.

#### Employing Technologies for OAC

Another trend that the government frames is the combination of technology and OAC. The advocation of technology for OAC was first introduced in the 13th FYP (2016–2020). During this period, “designing programs aimed at finding systematic technological solutions to address bottlenecks in old-age care” (Chap. 6, Sect. 1) was encouraged, suggesting that the government recognised the potential of technology in OAC. The government also proposed in the 14th FYP (2021–2025) to “develop age-friendly technologies and products, and smart old-age care services and other new business models” (Chap. 45, Sect. 3), and clarified the purpose of applying digital services to OAC, which is to ensure accessibility so that everyone can benefit (Chap. 16, Sect.1). The proposed utilisation of technology in OAC marked the beginning of a shift in the approach to caring for older adults from one that relies on human caregivers to one that makes use of technology as part of care provision. With the help of technology, older adults can maintain their independence for longer and promote their social connections, which aligns with the concept of active ageing (Lee et al., 2025).

#### Improving Pension Schemes

As highlighted in earlier sections, improving income security is an element of the Chinese understanding of OAC. All five FYPs highlighted the necessity of improving pension schemes. Table 3 gives an overview of the expanding pension schemes in China’s FYPs from 2001 to 2025.

**Table 3:** Texts related to improving pension schemes in FYPs

Document	Location	Content
The 10th FYP (2001–2005)	Chapter 18, Sect. 2	Expand the coverage of pension insurance and refine the basic pension insurance system for urban workers.
The 11th FYP (2006–2010)	Chapter 6, Sect. 4	Explore and establish a rural old-age insurance system.
	Chapter 39, Sect. 3	Expand the coverage of urban basic pension insurance.
The 12th FYP (2011–2015)	Chapter 33, Sect. 1	Achieve the full coverage of the new rural pension insurance scheme. Improve the pension insurance scheme for urban workers and non-working urban residents. Gradually promote the effective link between urban and rural pension schemes.
The 13th FYP (2016–2020)	Chapter 64, Sect. 1	Establish a mechanism for making appropriate adjustments to basic pensions.
The 14th FYP (2021–2025)	Chapter 49, Sect. 1	Pension insurance will cover the entire legally eligible population. Adjustments to the basic pension for urban workers will be more reasonable, leading to a raising pension standard for urban and rural residents.

The 10th (2001–2005), 11th (2006–2010), and 12th FYP (2011–2015) consistently focused on expanding pension insurance coverage, starting with urban workers and gradually extending to urban residents outside the workforce, and rural workers and residents. The rural pension scheme was established during the period of the 11th FYP (2006–2010). The three pension schemes - urban worker pension scheme, urban non-worker pension scheme, and rural pension scheme - were planned to be integrated, as seen in the 12th FYP (2011–2015). The aim of achieving fully integrated new pension scheme coverage was emphasised in the 14th FYP (2021–2025). Improvements in pension standards were advocated in the 13th FYP (2016–2020) and 14th FYP, which took economic development and rising prices into consideration. The pension schemes have been continually adjusted during the last 25 years, resulting in a pension regime that covers workers, urban residents without work and rural residents (Liu & Sun, 2016; Zhu & Walker, 2018). Ongoing reforms to the pension scheme are indicative of the Chinese government’s growing responsibility for the financial well-being of older adults, which explains why pensions have become the main source of income for older adults. The improved pension schemes reduce older adults’ financial dependency on families.

The sustainability of pensions was first put on the agenda of the 14th FYP (2021–2025). “The old-age insurance system will be improved to ensure the long-term balance of basic pension insurance funds” (Chap. 49, Sect. 1). The Chinese government has begun to realise the need and urgency to maintain a financially sustainable pensions system over an extended period. According to the Pension Fund Actuarial Report 2019–2060, China’s accumulated balance^1^ of basic pension funds may be depleted by 2044, with the active-to-retiree ratio projected to fall to 1 by 2060 (China Academy of Social Sciences & World Social Security Research Centre, 2025). This latest policy suggests that the government plays a role in managing and guarding the financially healthy pension schemes which in turn underpins the increasing economic independence of the older population from their families and lessens the need for family support (Cheung, 2023).

Overall, the FYPs indicate that the state has taken an increasingly active role in OAC. It has steadily expanded OAC provisions beyond those provided by families, including the development of the OAC systems, care services, technological applications and pension schemes. These policy developments expand older adults’ access to care services and strengthen their economic security, thereby helping to reduce their dependence on family, suggestive of emerging de-familialism.

### The Role of Individuals in OAC

#### Encouraging Private OAC Consumption

The consumption of private OAC services has been continually encouraged by the Chinese government. The 12th FYP (2011–2015) promoted the construction of public and private facilities for older adults, such as community day-care centres and nursing homes (Chap. 16, Sect. 3; Chap. 36, Sect. 4), focusing on the quantity of care facilities for older adults. The development of care facilities offers alternatives to family care and is indicative of a slight move in the direction of de-familialism in policymaking. Later, in the 13th FYP (2016–2020), the Chinese government sought to promote OAC consumption (Chap. 10, Sect. 1). A range of OAC products and services to meet the needs of older people are mentioned, and both the quantity and quality of OAC are emphasised, including customised OAC plans to match the needs of individuals (Chap. 61, Sect. 2; Chap. 65, Sect. 2). The development of new forms of OAC was encouraged during this period (Chap. 35, Sect. 2) such as the combination of travelling and OAC, where older adults stay in a warm region during the winter, engaging in tourism, cultural activities, and health care. The 13th FYP (2016–2020) also pays much attention to facilitating private investment in OAC and “eliminating varieties of discriminatory regulation”, such as a long and complicated approval process for private investment (Chap. 24, Sect. 3) and “removing restrictions on market access of foreign capital” (Chap. 49, Sect. 4). The 14th FYP advocated to “expand the supply of high-quality consumer goods, medium and high-end products and services in elder care”, “improve the quality of elder care products and services” (Chap. 12, Sect. 1), and “develop service consumption, promote the improvement and expansion of consumption in elder care” (Chap. 14, Sect. 1), especially the “elder-friendly technology products” and “smart elder care services” (Chap. 45, Sect. 3). These policy provisions contribute to increasing the supply and accessibility of OAC products and services. Smart technologies and old-age friendly products and services can empower older adults to live independently, reducing their reliance on caregivers and maintaining connections with others (Freeman et al., 2020; Yang & Du, 2021). The promotion of OAC consumption reflects both active ageing ideation and incipient de-familialisation.

#### Extending Working Lives

Working longer or raising the retirement age is associated with earning wages for a longer period and accumulation of pension entitlements. Three out of the five FYPs mapped out the policy path of extending working lives, chiming with active ageing ideation. The 12th FYP (2011–2015) characterised older people as “human resources” and promised to “develop and utilise human resources of the older population” (Chap. 26, Sect. 4). In the context of FYPs, human resources refer to the quality and quantity of the working population, which matters not only for private companies but also for the development of the national economy. The Chinese government acknowledges the value of skills, talents, and knowledge that older adults have obtained over the course of their lives. This point was elaborated upon in the 13th FYP (2016–2020). The Chinese government further promised to “raise the employability of older members of the workforce” and “gradually increase retirement age” (Chap. 65, Sect. 1). The reason for postponing retirement age, as suggested in the 13th FYP (Chap. 65, Sect. 1), is the decline in the working population. The retirement age before the recent reform for men was 60, women in blue-collar jobs 50, and women in white-collar jobs 55 since the 1950 s, which was low compared to the life expectancy of 75.8 in 2013 and 77.6 in 2021 (WHO, 2024). The 14th FYP (2021–2025) further proposed to raise the “statutory retirement age” due to longer life expectancy, rapid population ageing, increased years spent in education, and changes in labour force structure (Chap. 45, Sect. 3). This decision to gradually raise the statutory retirement age has been adopted since 2025, and people at retirement age can voluntarily apply for a further extension (National People’s Congress, 2024). These policy directions match active ageing ideation that centres around longer working lives.

#### Improving Personal Pension Planning

As discussed earlier, pensions and family support remain the main sources of income for Chinese older adults. Rapid population ageing had led to a sharp rise in pensions expenditure in China, which challenges the fiscal sustainability of the pay-as-you-go pension system (Peng & Song, 2021; Wang & An, 2018). As one of the main sources of retirement income, pension planning is seen as providing a solid financial foundation for individuals to live independently and reduce their economic dependence on adult children (Su et al., 2022), hence aligning with both active ageing (ability to work, save, consume and remain independent) and de-familialism (independence from family). The FYPs introduced reforms of the social security system, including the “multi-pillar old-age insurance system”. It comprises basic old age insurance, enterprise annuity, and commercial and personal pension insurance, complementing each other. The Chinese government stresses the importance of individual financial planning for later life in both the 13th and 14th FYPs. The 13th FYP (2016–2020) launched tax-deferred pension insurance (Chap. 14, Sect. 1), and the 14th Five-Year Plan (2021–2025) promotes the implementation of personal pension insurance (Chap. 49, Sect. 1). Thus, the Chinese government partly transfers the responsibility of financing pensions to individuals (Li et al., 2021). In the government’s vision, individuals would actively enrol in personal pension insurance. However, after the implementation of personal pension insurance in 2022, only 9 million people had participated in this scheme by the end of March 2023 (Wang, 2023). The tendency towards promote commercial and personal pension insurance indicates that individuals are expected to take greater responsibility for financially preparing for retirement.

In general, the FYPs incorporate growing expectations of individual responsibility for well-being in later life, notably through private OAC consumption, prolonged working lives, and personal pension planning. These policy directions resemble key ideas associated with active ageing, particularly through their emphasis on economic participation and self-reliance in preparing for and financing OAC.

## Findings

### The Role of Families in OAC

### Supporting Family Care

The amount of text on the role of the family in OAC was limited, but where it featured, the text strongly emphasised the family’s role in three out of five documents. In the 10th FYP (2001–2005) on developing social security system, the Chinese government stated that it “encourages family care for older adults” (Chap. 13, Sect. 8). This abstract advocation presents the government’s expectation that family members should take the responsibility for caring for older relatives, which aligns with traditional OAC ideation and reconfirms the importance of family care for older people. At this stage, there were no policies that directly supported or discouraged family care and the state endorsed the family’s assumed care function, adhering to the logic of implicit familialism. The next two FYPs of 2006–2010 and 2011–2015 did not pay attention to family care for older people.

This situation has changed since the year 2016. Instead of simply expecting and exhorting families to take care of older people, the 13th FYP (2016–2020) turned to supporting family care through policy. In the 13th and 14th FYPs, the Chinese government promised to develop policies to support family caregiving in the sections outlining responses to population ageing:

“We will … establish policies to support family care for older adults and enhance the function of family in caring for older people”. (The 13th FYP, Chap. 64, Sect. 3)

“Efforts will be made to … support family care for older adults”. (The 14th FYP, Chap. 45, Sect. 3)

The focus on family care in FYPs has shifted from expecting family members to care for older adults to making efforts to support family care. For example, China announced a reduction in individual income tax for those who have ageing parents to care for in 2018 and a further reduction in 2023 (The State Council Information Office, 2023). These policy developments continue to position the family as the primary provider of OAC while introducing state support that facilitates the fulfilment of family caregiving responsibilities. As such, they resemble the core characteristics of Leitner’s explicit familialism.

## Discussion

The aim of this article was to examine changes in the role of families, the state, and individuals in OAC in formal policy planning process in China, utilising the concepts of active ageing and (de-)familialism to gauge the direction of policymaking.

The family has been relied on until now and is still expected to be a central source of care for older adults in China. The Chinese government continues to utilise policy tools to reinforce the responsibility of families for OAC, both implicitly and explicitly. Earlier policy plans analysed here primarily emphasised family care duties without providing supportive interventions. However, more recent FYPs have introduced interventions to assist families in caregiving roles, such as fiscal support through tax reduction. The legal obligation of caring for older adults and tax supports introduced recently reaffirm the importance of family in OAC, further sustaining older adults’ reliance on their families. This suggests that policy priorities in the most recent FYPs align with Leitner’s (2003) framework of explicit familialism, wherein the state strengthens and supports family caregiving duties. However, the policy approach reflected in the FYPs relies heavily on an indirect form of support, namely tax relief, whereas the European policy examples discussed by Leitner (2003) commonly feature direct cash transfers and support services, including care allowances and carers’ allowances, as well as legislated care leaves. Nonetheless, indirect tax relief provides only limited support in meeting the needs of family caregivers (Bai & Li, 2020). The decreasing family size and the rapid population ageing in China challenges the caring function of the family (Cheung, 2019; Hu & Peng, 2018; Gong, 2020). Urbanisation and internal migration further limit families’ capability to care for older adults since adult children reside far from their parents (Chen et al., 2014; He & Ye, 2014; Liu & Cook, 2020; Zhao et al., 2020). As the demographic changes and social transformations have made it harder for families to take full responsibility for OAC, the expanded role of other stakeholders, including the government, has become a more prominent feature in China’s policy.

Across the five FYPs analysed, the Chinese government has assumed growing responsibility for OAC. It has expanded care provision, developed community-based and institutional care, improved pension schemes, and promoted the use of OAC technologies. These policy developments increase the availability of formal care and provide older adults with financial support beyond the family. However, these policy orientations do not seek to replace families as the primary providers of care. Instead, these OAC services are primarily intended to support and complement family care, with home- and community- based care remaining the advocated care arrangement and institutional care positioned as supplementary. The expansion of pension schemes strengthens older adults’ economic security and increases their capacity to finance their own care (Li et al., 2021), thereby reducing financial dependence on family members and providing the potential for individuals to self-organise their care. Similarly, smart care technologies enable older adults to remain living at home while maintaining independence. Nevertheless, the family still takes the main responsibility in care provision (Chappell & Funk, 2012; Cui & Du, 2021; Lee, 2018; Song & Ji, 2020) and the formal OAC services have not been widely accepted (Ma et al., 2019; Su et al., 2022). Together, these policy directions increase the provision of OAC and complement existing family support. Therefore, the policy trajectory in the FYPs reflects an incipient de-familialising tendency within a predominately familialist policy framework.

The general trend in the FYPs is that individuals are expected to assume greater responsibility for OAC through working longer, personal pension planning and private OAC consumption. Working longer is a central part of active ageing, with attendant expectations that individuals stay socially connected and economically independent. Personal pension planning enhances individuals’ role in securing their own financial well-being in old age. Furthermore, older adults are encouraged to spend on OAC products and services, in the context of the government promoting the development of the OAC market. These policy directions in the FYPs align with the ideation of active ageing promoted by Western welfare states, as individuals’ economic independence and their personal responsibility for OAC is highlighted in both the Western and Chinese contexts.

## Conclusion

The shift of OAC responsibilities in China is indicative of a dynamic interplay among families, the government, and individuals. China’s OAC policy supports family care and imposes the legal obligations to support parents, in line with explicit familialism. With continued reliance on family care, family caregivers are struggling to shoulder OAC responsibilities due to demographic and social changes. The Chinese government has responded to this by improving old-age income security, developing the OAC market, and utilising technological innovations, thereby fostering a model that increasingly leans towards active ageing and incipient de-familialisation. Individuals are encouraged to assume greater financial responsibility for their later life (in line with active ageing) by strengthening their retirement security and consuming OAC products and services (de-familialism). The sustainability of pension funds, the advocacy of personal pension planning, and the encouragement on OAC consumption have grown in importance in China’s OAC policy. The combination of technology and OAC is newly emerging and is portrayed as having potential to reduce older adults’ reliance on caregivers in daily activities. Manifestations of active ageing and incipient de-familialisation therefore feature in the general direction of China’s OAC policy as evidenced by our analysis of the FYPs over the last quarter century. Further research should investigate the adaptation of technologies and other aspects of the Chinese version of active ageing, and the evolving role of families both in policy and practices of care. In combination, the move towards more explicit familialism and active ageing points in the direction of parallels with policy development in other parts of the world (cf. Kodate & Timonen, 2017), and such potential convergence merits further interrogation.

## Data Availability

The data used in this study are publicly available Five-Year Plans (Chinese) from the official website of the Chinese government. These documents can be accessed at https://www.gov.cn/gongbao/content/2001/content_60699.htm; https://www.gov.cn/gongbao/content/2006/content_268766.htm; https://www.gov.cn/2011lh/content_1825838.htm; https://www.gov.cn/xinwen/2016-03/17/content_5054992.htm; https://www.gov.cn/xinwen/2021-03/13/content_5592681.htm.

## Footnotes

1 Accumulated balance reflects net assets from cumulative contributions minus expenditures, covering both individual and pooling accounts.
